# Upper Limb Stroke Rehabilitation Using Surface Electromyography: A Systematic Review and Meta-Analysis

**DOI:** 10.3389/fnhum.2022.897870

**Published:** 2022-05-20

**Authors:** Maria Munoz-Novoa, Morten B. Kristoffersen, Katharina S. Sunnerhagen, Autumn Naber, Margit Alt Murphy, Max Ortiz-Catalan

**Affiliations:** ^1^Department of Clinical Neuroscience, Institute of Neuroscience and Physiology, Sahlgrenska Academy, University of Gothenburg, Gothenburg, Sweden; ^2^Center for Bionics and Pain Research, Mölndal, Sweden; ^3^Department of Orthopaedics, Institute of Clinical Sciences, Sahlgrenska Academy, University of Gothenburg, Mölndal, Sweden; ^4^Section of Neurocare, Sahlgrenska University Hospital, Gothenburg, Sweden; ^5^Department of Occupational Therapy and Physiotherapy, Sahlgrenska University Hospital, Gothenburg, Sweden; ^6^Operational Area 3, Sahlgrenska University Hospital, Mölndal, Sweden; ^7^Department of Electrical Engineering, Chalmers University of Technology, Gothenburg, Sweden

**Keywords:** electromyography, stroke, upper limb function, Fugl-Meyer Assessment, biofeedback, paresis

## Abstract

**Background:**

Upper limb impairment is common after stroke, and many will not regain full upper limb function. Different technologies based on surface electromyography (sEMG) have been used in stroke rehabilitation, but there is no collated evidence on the different sEMG-driven interventions and their effect on upper limb function in people with stroke.

**Aim:**

Synthesize existing evidence and perform a meta-analysis on the effect of different types of sEMG-driven interventions on upper limb function in people with stroke.

**Methods:**

PubMed, SCOPUS, and PEDro databases were systematically searched for eligible randomized clinical trials that utilize sEMG-driven interventions to improve upper limb function assessed by Fugl-Meyer Assessment (FMA-UE) in stroke. The PEDro scale was used to evaluate the methodological quality and the risk of bias of the included studies. In addition, a meta-analysis utilizing a random effect model was performed for studies comparing sEMG interventions to non-sEMG interventions and for studies comparing different sEMG interventions protocols.

**Results:**

Twenty-four studies comprising 808 participants were included in this review. The methodological quality was good to fair. The meta-analysis showed no differences in the total effect, assessed by total FMA-UE score, comparing sEMG interventions to non-sEMG interventions (14 studies, 509 participants, SMD 0.14, P 0.37, 95% CI –0.18 to 0.46, I^2^ 55%). Similarly, no difference in the overall effect was found for the meta-analysis comparing different types of sEMG interventions (7 studies, 213 participants, SMD 0.42, P 0.23, 95% CI –0.34 to 1.18, I^2^ 73%). Twenty out of the twenty-four studies, including participants with varying impairment levels at all stages of stroke recovery, reported statistically significant improvements in upper limb function at post-sEMG intervention compared to baseline.

**Conclusion:**

This review and meta-analysis could not discern the effect of sEMG in comparison to a non-sEMG intervention or the most effective type of sEMG intervention for improving upper limb function in stroke populations. Current evidence suggests that sEMG is a promising tool to further improve functional recovery, but randomized clinical trials with larger sample sizes are needed to verify whether the effect on upper extremity function of a specific sEMG intervention is superior compared to other non-sEMG or other type of sEMG interventions.

## Introduction

Stroke is one of the leading causes of disability in adults (Feigin et al., 2017). Approximately 50 to 70% of individuals with stroke demonstrate upper limb impairment in the acute phase (Persson et al., 2012; Raffin and Hummel, 2018), and only 5 to 20% regain full upper limb dexterity 6 months after the onset of stroke (Nijland et al., 2010). Upper limb impairment limits activities of daily living and participation in different social contexts and physical environments (Foley et al., 2019).

Upper limb rehabilitation is crucial to maximize outcomes and decrease disability (Pollock et al., 2013; Hatem et al., 2016). Along with traditional clinical interventions, several technologies such as neuromuscular stimulation, invasive and non-invasive brain stimulation, robotic devices, virtual reality gaming, and electromyography (EMG) exist to enhance recovery after stroke (Pollock et al., 2013; Hatem et al., 2016). Each technology has advantages and disadvantages and relies on different rehabilitation approaches to improve upper limb function.

Surface EMG (sEMG), in which electrodes placed over the skin record the electrical activity of a muscle or group of muscles, has been used for neurorehabilitation for more than five decades (Criswell, 2010; Merletti and Farina, 2016; Campanini et al., 2020). sEMG can be applied as an assessment to evaluate muscle activation patterns or as a tool to complement and enhance different neuromuscular rehabilitation interventions (Criswell, 2010; Merletti and Farina, 2016; Campanini et al., 2020; Mcmanus et al., 2020). sEMG is an objective method that provides real-time information on muscle activity in terms of timing, location, and contraction intensity (Criswell, 2010; Merletti and Farina, 2016; Mcmanus et al., 2020). One advantage of the sEMG is that it can record muscle activity even when no visible movement or palpable muscle activity is present (Campanini et al., 2020; Cappellini et al., 2020), which widens the possible application areas (Criswell, 2010; Merletti and Farina, 2016; Campanini et al., 2020; Mcmanus et al., 2020).

In stroke, sEMG has been used for upper limb rehabilitation since the beginning of the 90s (Merletti and Farina, 2016). The visual and/or auditory biofeedback on muscle activity provided by an sEMG system has been used to enhance motor function and facilitate learning towards a more effective use of the affected limb (Schleenbaker and Mainous, 1993; Woodford and Price, 2007; Giggins et al., 2013). In addition, sEMG has been applied to trigger neuromuscular electrical stimulation (NMS) for specific target muscle groups to promote upper limb function in stroke (Takeda et al., 2017; Monte-Silva et al., 2019). Furthermore, electromechanical and robot-assisted arm training using sEMG signals to drive robotic devices, such as an exoskeleton or active orthosis, has shown to improve upper limb function and performance in activities of daily living (Norouzi-Gheidari et al., 2012; Mehrholz et al., 2015; Hameed et al., 2020). The feedback provided by sEMG can increase awareness, self-regulation, precision, and control of muscle contraction in real-time, which might improve compliance and motivation (Criswell, 2010; Merletti and Farina, 2016; Mcmanus et al., 2020).

Taking the large variety of clinical application areas of sEMG, more knowledge is needed to better understand the benefits, possibilities, and potential clinical effects of the different types of sEMG interventions on motor recovery after stroke (Campanini et al., 2020; Mcmanus et al., 2020). Previous reviews and meta-analyses have exclusively been dedicated to one specific type of sEMG intervention (Schleenbaker and Mainous, 1993; Bolton et al., 2004; Woodford and Price, 2007; Meilink et al., 2008; Norouzi-Gheidari et al., 2012; Basteris et al., 2014; Mehrholz et al., 2015; Eraifej et al., 2017; Takeda et al., 2017; Monte-Silva et al., 2019; Yang et al., 2019; Hameed et al., 2020), and summarized knowledge on the effect of different types of sEMG-driven interventions on upper limb function in stroke is limited. Thus, the aim of this systematic review and meta-analysis is to synthesize existing evidence on the effect of different sEMG-driven interventions on upper limb impairment in people with stroke and identify which type of sEMG intervention could be beneficial for this purpose.

## Materials and Methods

The present systematic review and meta-analysis was registered in the PROSPERO database prior analysis (CRD42021243372), and the PRISMA 2020 statement was followed (Page et al., 2021).

### Search Strategy

One of the authors (MM-N) conducted an online search in PubMed, Scopus, and PEDro databases, including relevant articles in English published between January 2000 and May 2021. The search terms used are shown in Table 1. In addition, the references of identified relevant papers and existing systematic reviews and meta-analyses in the field were screened (Schleenbaker and Mainous, 1993; Bolton et al., 2004; Woodford and Price, 2007; Meilink et al., 2008; Norouzi-Gheidari et al., 2012; Basteris et al., 2014; Mehrholz et al., 2015; Eraifej et al., 2017; Takeda et al., 2017; Monte-Silva et al., 2019; Yang et al., 2019; Hameed et al., 2020; Doumas et al., 2021).

**TABLE 1:** Terms and search strategy.

Main Term	Keyword and/or MESH term
1.Stroke	*“cerebrovascular disorders”* OR *“stroke”* OR *“stroke rehabilitation”* OR “*CVA*” OR *“cerebrovascular*” OR “*cerebral vascular*”
2.Electromyography	“*EMG*” OR *“electromyography”* OR *“myography”*
3.Control mode	“*control*” OR “*trigger*” OR *“drive”* OR *“feedback”* OR *“biofeedback”*
4.Upper extremity	*“upper extremity”* OR *“elbow”* OR *“arm”* OR *“forearm”* OR *“hand”* OR *“wrist”* OR *“finger*
5.Hemiparesis	*“plegia”* OR *“paresis”*
**Final search:**	1 AND 2 AND 3 AND 4 AND 5

*CVA, cerebrovascular accident; EMG, Electromyography.*

### Selection Criteria

Studies were eligible for inclusion if participants were older than 18 years and had an upper limb motor impairment due to stroke. Only randomized control trials (RCT) using sEMG interventions to improve upper limb function were included. The sEMG signal needed to be obtained from the upper limb, and the protocol was required to explicitly state the number of sessions, time, and frequency of treatment. Fugl-Meyer Assessment of Upper Extremity (FMA-UE) (Fugl-Meyer et al., 1975) needed to be one of the included outcomes. The FMA-UE is a recommended assessment for upper limb sensorimotor function in stroke trials and is considered as a gold standard (Fugl-Meyer et al., 1975; Gladstone et al., 2002; Hernández et al., 2019). Studies were excluded if sEMG signal recording was only used as an assessment instead of an intervention tool or if the sEMG intervention was not the study primary focus. For the studies where the FMA-UE total score was not specified, the corresponding author was contacted. Studies were excluded from the meta-analysis if it was not possible to obtain the total FMA-UE score.

One reviewer performed the screening for title and abstract for relevance (MM-N). Following, full texts of all potentially pertinent articles were reviewed by two authors (MM-N, MBK). Then, the two reviewers’ opinions were compared, and any disagreement was resolved by a third reviewer (MAM).

### Data Extraction

The data from the included studies were extracted by one reviewer (MM-N) and independently checked by a second reviewer (MBK). The extracted data included: study characteristics (study design, year published, inclusion/exclusion criteria, and sample size), participant characteristics (stroke onset, upper limb impairment), interventions (type of sEMG intervention, sEMG electrode site, and training description), treatment protocol (number of sessions, frequency, session duration and follow-up times), setting (hospital, laboratory, or home), outcomes (FMA-UE scores on control and experimental group at all time points), main results and conclusions.

Time since stroke was classified as acute (< 7 days), subacute (7 days to 6 months), and chronic (≥ 6 months) (Hatem et al., 2016). The upper limb impairment was categorized according to the baseline mean value of the total FMA-UE scores reported in the articles. FMA-UE score < 31 was defined as “severe”, 32-57 as “moderate” and 58-66 as “mild” impairment (Pang et al., 2006; Persson et al., 2015; Bustrén et al., 2017).

The sEMG interventions used in different studies were categorized into subgroups according to their type of application: sEMG-triggered neuromuscular stimulation (sEMG-NMS), sEMG providing visual and/or auditory biofeedback (sEMG-BFB), sEMG-driven robotic device (sEMG-RT), or a combination of those. The control or comparison interventions that did not include sEMG were categorized as non-sEMG interventions (e.g., conventional care, cyclic NMS, passive motion robot).

For studies comparing different sEMG interventions protocols with each other, the interventions were categorized as Intervention 1 when the sEMG interventions was utilized alone, and as Intervention 2 when the sEMG intervention was combined with another training modality (e.g., mirror therapy, task-oriented training, mental imagery).

### Methodological Quality Assessment

The methodological quality of the randomized controlled trials was assessed independently by two reviewers (MBK, MM-N) using the Physiotherapy Evidence Database (PEDro) scale. This scale is used to evaluate the quality and potential risk of bias of randomized controlled trials (Maher et al., 2003; Moseley et al., 2019). A third reviewer (MAM) was consulted in case of any discrepancy. PEDro scores of 9 to 10 were considered as “excellent,” 6 to 8 as “good”, 4 to 5 as “fair”, and below 4 as “poor” quality (Maher et al., 2003; Moseley et al., 2019). Only studies with a PEDro score 4 and more were included for the meta-analysis.

### Data Analysis

The included studies comparing sEMG interventions with non-sEMG interventions and studies comparing sEMG interventions with another sEMG intervention protocol were summarized and analyzed separately. For studies comparing more than two intervention protocols, each comparison was included independently under the respective type of sEMG intervention.

In the meta-analysis, the effect size of each study was determined using the Hedges’ g measure (Harrer et al., 2021). In case a study did not provide the standard deviation (SD) of the change from baseline, the data were imputed using the Cochrane correlation method (Higgins et al., 2019). The standardized mean difference (SMD) with 95% confidence intervals was used for the pooled effect using the FMA-UE total score. Statistical heterogeneity was calculated using the I^2^ test (Higgins et al., 2019; Deeks et al., 2021; Harrer et al., 2021). Heterogeneity values between 30 and 60% suggest moderate heterogeneity, 50%-90% might represent substantial heterogeneity, and 75-100% represent considerable heterogeneity (Higgins et al., 2019). A random-effect model was used (Higgins et al., 2019; Deeks et al., 2021; Harrer et al., 2021). Sub-group analyses among the different types of sEMG intervention were also performed. The meta-analysis and forest plots were produced with R software and R studio statistical program (version R-4.0.5) (Harrer et al., 2021).

## Results

### Included Studies and Methodological Quality Assessment

**FIGURE 1:**
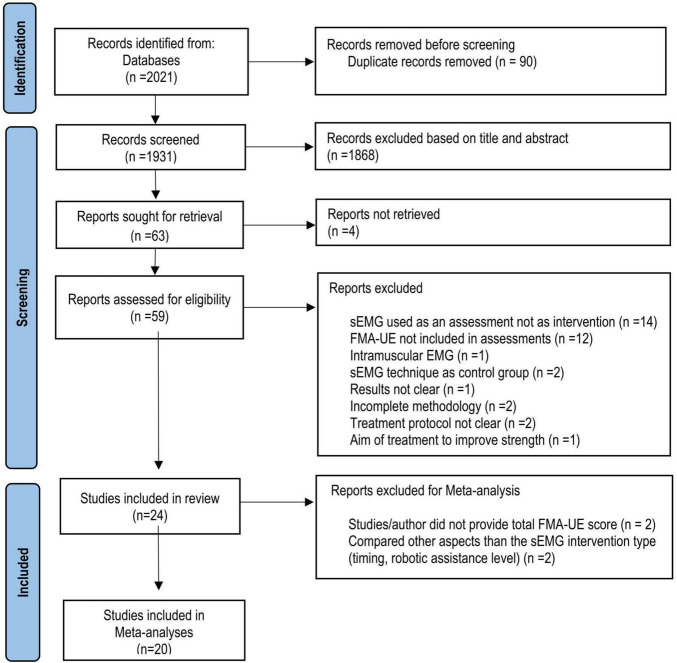
Flow chart (PRISMA) of study selection. RCT, Randomized clinical trial; sEMG; surface electromyography, FMA-UE, Fugl-Meyer Assessment for the upper extremity.

In total, 2021 articles were identified using the search strategy (Figure 1). After the screening for eligibility, 24 studies from 10 countries were included in the systematic review. Of those, 14 studies comparing sEMG with non-sEMG interventions and 7 studies comparing sEMG interventions with another sEMG intervention protocol were included in the meta-analysis. One study with three intervention groups (Page et al., 2020) was included in the analysis of sEMG compared with non-sEMG as well as in the analysis of sEMG intervention compared to another sEMG intervention. Four studies were not included in the meta-analysis, of which two did not provide the total FMA-UE score (Hu et al., 2009, 2015), and two used the same type of sEMG intervention in both groups and compared other aspects of the intervention (immediate versus delayed protocol and robotic assistance applied at upper arm versus assistance applied at wrist level, respectively) (Kojima et al., 2014; Qian et al., 2019). According to the PEDro scale 18 RCTs showed good and 6 RCTs fair methodological quality (Table 2).

**TABLE 2:** Methodological quality assessment of included studies utilizing PEDro scale.

Authors	Items of PEDro scale											Total score
	1. Eligibility criteria	2. Random allocation	3. Concealed allocation	4. Baseline comparability	5. Blind subjects	6. Blind therapists	7. Blind assessors	8. Adequate follow-up	9. Intention-to-treat analysis	10. -group comparisons	11. Point estimates and variability	
Hemmen and Seelen, 2007	1	1	1	0	0	0	1	1	1	1	1	**7**
de Kroon and IJzerman, 2008	1	1	1	0	0	0	1	1	1	1	1	**7**
Hu et al., 2009	1	1	0	1	0	0	1	1	0	1	1	**6**
Shindo et al., 2011	1	1	0	1	0	0	1	0	0	1	1	**5**
De Araújo et al., 2011	0	0	0	1	0	0	0	1	1	1	1	**5**
Boyaci et al., 2013	1	1	1	1	0	0	1	1	1	1	1	**8**
Cordo et al., 2013	1	1	0	0	0	0	1	1	0	1	1	**5**
Page et al., 2013	1	1	0	1	0	0	1	1	1	1	1	**7**
Singer et al., 2013	1	1	0	1	0	0	0	1	0	1	1	**5**
Kojima et al., 2014	1	1	0	1	0	0	0	1	1	1	1	**6**
Hu et al., 2015	1	1	0	1	0	0	1	1	1	1	1	**7**
Kim et al., 2016	1	1	0	1	0	0	0	1	1	1	1	**6**
Wilson et al., 2016	1	1	1	1	0	0	1	1	1	1	1	**8**
Kwakkel et al., 2016	1	1	1	1	0	0	1	1	1	1	1	**8**
Amasyali and Yaliman, 2016	0	1	0	1	0	0	1	1	0	1	1	**5**
Schick et al., 2017	1	1	1	1	0	0	1	1	0	1	1	**7**
Qian et al., 2017	1	1	0	1	0	0	1	1	1	1	1	**7**
Zhou et al., 2017	1	1	0	1	0	0	1	1	0	1	1	**6**
Qian et al., 2019	1	1	1	1	0	0	1	1	1	1	1	**8**
Mugler et al., 2019	1	1	0	1	0	0	1	1	0	1	1	**6**
Park, 2020	1	1	1	1	0	0	1	1	1	1	1	**8**
Huang et al., 2020	1	1	0	1	0	0	1	1	1	1	1	**7**
Obayashi et al., 2020	1	0	0	1	0	0	0	1	1	1	1	**5**
Page et al., 2020	1	1	1	1	0	0	1	1	1	1	1	**8**

*PEDro item 1 evaluates the external validity, and it is not included in the sum of the total score (Maher et al., 2003; Moseley et al., 2019).

### Characteristics of the Included Studies

Table 3 summarizes the studies that compared sEMG interventions with non-sEMG interventions, and Table 4 shows the studies that compared the sEMG interventions alone with the sEMG interventions combined with another training modality. One study (Page et al., 2020), reporting comparisons both with a non-sEMG intervention and with another sEMG intervention, was included in both tables.

**TABLE 3:** Characteristics of the included studies comparing sEMG intervention with non-sEMG intervention.

Author (year)	n	Age (years) mean ± SD	Stroke stage	UE impairment	sEMG intervention (E)	Non-sEMG intervention (NE)	Protocol treatment	Setting	Improvement of UE impairment (FMA-UE)	
									All groups improve	Significant difference between groups
Hemmen and Seelen, 2007	E:14	62.1 ± 12.7	Subacute	Moderate	sEMG-NMS + movement imagery	Conventional ES	60 sessions, 5x/w, 30 min	Hospital	Yes	No
	NE:13	60.7 ± 12.3								
de Kroon and IJzerman, 2008	E:11	57.4 ± 8.0	Chronic	Moderate to severe	sEMG-NMS	Cyclic ES	≤ 126 sessions, 7x/w, 30 min, 3x/day	Home	No significantly	No
	NE:10	60.6 ± 10.9								
*Hu et al., 2009	E:15	49.2 ± 14.7	Chronic	Moderate	sEMG-RT	Passive motion robot	20 sessions, 3-5 x/w, time N/R	Hospital	No, only E.	Yes (favoring E, shoulder/elbow)
	NE:12	53.3 ± 10.4								
Shindo et al., 2011	E:10	58.2 ± 18.6	Subacute	Moderate to severe	sEMG-NMS + wrist splint (HANDS)	Wrist splint	21 sessions, 7x/w, 8 hours	Home	Yes	Yes (favoring E, wrist/hand)
	NE:10	57.9 ± 9.7								
De Araújo et al., 2011	E:6	42.8 ± 14.0	Chronic	Moderate to severe	sEMG electro-mechanical orthosis	Usual care	24 sessions, 3x/w, 50 min	Lab	Yes	Yes (favoring E wrist/hand)
	NE:6	52.6 ± 17.8								
Boyaci et al., 2013	E1:11	56.1 ± 6.8	Subacute and chronic	Moderate to severe	sEMG-NMS + sEMG-BFB	NE1: Passive NMS	15 sessions, 5 x/w, 45 min	Hospital	No, only E and NE1.	Yes (favoring E. No difference between E and NE1).
	NE1:10	64.4 ± 9.5				NE2: Sham stimulation				
	NE2:10	57.6 ± 16.4								
Cordo et al., 2013	E:21	54.0 ± 12.0	Chronic	Severe	sEMG-BFB + Robot-assisted + muscle vibration	Robot-assisted movement + muscle vibration + torque	30 sessions, 10-12 weeks, 30 min	Hospital	Yes	No
	NE:22	57.0 ± 10.0								
Page et al., 2013	E:8	59.0 ± 12.9	Chronic	Severe	sEMG-RT + repetitive task-specific	Usual care repetitive task-specific	24 sessions, 3 x/w, 30 min	Lab	No significantly	No
	NE:8	58.5 ± 9.5								
Wilson et al., 2016	E:37	58.6 ± 13.1	Subacute	Severe	sEMG-NMS	NE1: Cyclic NMS	80 sessions, 5 x/w, 2x/day, 40 min	Home	Yes	No
	NE1:35	55.0 ± 16.1				NE2: Sensory stimulation				
	NE2:37	55.8 ± 16.1								
Kwakkel et al., 2016	E:50	58.9 ± 11.6	Subacute	Severe	sEMG-NMS + sEMG-activity visualized in a computer game	Usual care	E: 30 sessions, 5 x/w,2x/day, 30 min	Hospital	No significantly	No
	NE:51	58.5 ± 11.8					NE: 15 sessions, 5 x/w, 30 min			
Amasyali and Yaliman, 2016	E:7	51.2 ± 12.2	Subacute and chronic	Moderate	sEMG-NMS	NE1: MT	E-NE1: 15 sessions, 3 x/w, 30 min	Hospital and home	No, only E and NE1.	No
	NE1:7	58.7 ± 10.1								
	NE2:7	65.3 ± 9.0				NE2: Conventional physiotherapy alone	NE2: 15 sessions, 3 x/w, 2 hours			
Qian et al., 2017	E:14	54.6 ± 11.3	Subacute	Severe	sEMG-NMS + sEMG-RT	Conventional physiotherapy	20 sessions, 5 x/w, 40 min	Hospital	Yes	Yes (favoring E, wrist/hand)
	NE:10	64.6 ± 3.4								
Zhou et al., 2017	E:18	50.9 ± 13.8	Subacute	Severe	sEMG-NMS bridge: sEMG from non-paretic limb	Cyclic NMS	10 sessions, 2 x/w, 20 min	Hospital	Yes	Yes (favoring E)
	NE:18	56.9 ± 10.0								
Obayashi et al., 2020	E:8	71.0 ± 13.8	Acute and subacute	Severe	sEMG-NMS + Usual care	Usual care	3 to 35 sessions, 5 x/w, 15 to 20 min	Hospital	Yes	No
	NE:9	72.3 ± 10.7								
Page et al., 2020	E1:8	52.8 ± 11.3	Chronic	Severe	E1: sEMG-RT + repetitive task-specific	Repetitive task-specific practice	24 sessions, 3 x/w, 60 min	Hospital	Non	No
	E2:14	55.7 ± 9.2			E2: sEMG-RT					
	NE:9	57.2 ± 7.6								

s-EMG, surface electromyography; E, sEMG intervention group; NE, non-sEMG intervention group; UE, upper extremity, NMS, neuromuscular stimulation; ES, electrostimulation; sEMG-NMS, sEMG triggered neuromuscular stimulation; sEMG-RT, sEMG driven robot therapy; BFB, Biofeedback; MT, Mirror therapy; FMA-UE, Fugl-Meyer Assessment for the upper extremity; N/R, No Reported; +, combined with; s, total sessions; x/w, times per week; Lab, laboratory; (*), not included on meta-analysis.

**TABLE 4:** Characteristics of the included studies comparing sEMG intervention with another sEMG intervention.

Author (year)	n	Age (years) mean ± SD	Stroke stage	UE impairment	Intervention 1 (Int 1)	Intervention 2 (Int 2)	Protocol treatment	Setting	Improvement on FMA-UE	
									All groups improve	Significant difference between groups
Singer et al., 2013	Int 1:10	68.0 ± 16.4	Chronic	Moderate to severe	sEMG-NMS + unilateral task specific practice	sEMG-NMS + bilateral task specific practice	30 sessions, 6-7 x/w, 30 min 2x/day	Home	Yes	No
	Int 2:11	68.6 ± 9.0								
*Kojima et al., 2014	Int 1:7	67.7 ± 15.5	Subacute	Moderate	Delayed group: PT + OT alone for the first 4 weeks, followed by 4 weeks of sEMG-NMS + MT + PT + OT	Immediate group: sEMG-NMS + MT + PT + OT for the first 4 weeks, followed by 4 weeks of PT + OT alone	20 sessions, 5 x/w, 20 min, 2x/day (sEMG-NMS stage)	Hospital	Yes	Yes (favoring Int 2)
	Int 2:6	70.7 ± 10.3								
*Hu et al., 2015	Int 1:15	49.2 ± 14.7	Chronic	Moderate	sEMG-RT	sEMG-NMS + sEMG-RT	20 sessions, 3 to 5 x/w, time N/R	Hospital	Yes	Yes (favoring Int 2)
	Int 2:11	45.6 ± 11.4								
Kim et al., 2016	Int 1:10	47.5 ± 14.4	Chronic	Moderate	sEMG-NMS	sEMG-NMS + task-oriented training	20 sessions, 5 x/w, 20 min	Hospital	Yes	Yes (favoring Int 2)
	Int 2:10	48.9 ± 10.1					20 sessions, 5 x/w, 30 min			
Schick et al., 2017	Int 1:17	63.0 ± 11.5	Subacute	Severe	sEMG-NMS bilateral	sEMG-NMS bilateral + MT	15 sessions, 3 x/w, 30 min	Hospital	Yes	No
	Int 2:15	62.0 ± 19.6								
*Qian et al., 2019	Int 1:15	57.7 ± 5.9	Chronic	Moderate to severe	sEMG-NMS + sEMG-RT support sleeve	sEMG-NMS + sEMG-RT support hand	20 sessions, 3 to 5 x/w, 60 min	Lab	Yes	Yes (favoring Int 2, wrist/hand)
	Int 2:15	57.3 ± 8.8								
Mugler et al., 2019	Int 1a:12	58.5 ± 19.4	Chronic	Severe	*sEMG-BFB, isometric 60 min	sEMG-BFB, movement-based training 90 min	6 sessions, 2 x/w, 60 or 90 min depends on protocol	Lab	Yes	No
	Int 1b:11	60.0 ± 7.2			sEMG-BFB, isometric 90 min					
	Int 2:9	56.8 ± 8.1								
Park, 2020	Int 1:34	65.7 ± 6.0	Chronic	Severe	sEMG-NMS	sEMG-NMS + mental imagery	30 sessions, 5 x/w, 30 min	Hospital	Yes	No
	Int 2:34	66.8 ± 6.5								
Huang et al., 2020	Int 1:15	60.0 ± 6.8	Chronic	Severe	sEMG-RT	sEMG-NMS + sEMG-RT	20 sessions, 3 to 5 x/w, 30 min	N/R	Yes	Yes (Int 2, favoring shoulder and wrist/hand)
	Int 2:15	57.3 ± 9.1								
Page et al., 2020	Int 1:14	55.7 ± 9.2	Chronic	Severe	sEMG-RT	sEMG-RT + repetitive task-specific	24 sessions, 3 x/w, 60 min	Hospital	Non	No
	Int 2:8	52.8 ± 11.3								

s-EMG, surface electromyography; Int, intervention group; UE, upper extremity, NMS, neuromuscular stimulation; sEMG-NMS, sEMG triggered neuromuscular stimulation; sEMG-RT, sEMG- driven robot therapy; BFB, Biofeedback; MT, Mirror therapy; FMA-UE, Fugl-Meyer Assessment for the upper extremity; N/R, Not Reported; +, combined with; s, total sessions; x/w, times per week; x/day, times per day; (*), not included on meta-analysis.(Intervention 1, study group utilized only sEMG modality/is on their rehabilitation protocol. Intervention 2, study group combine sEMG-driven intervention with another training modality).

**FIGURE 2:**
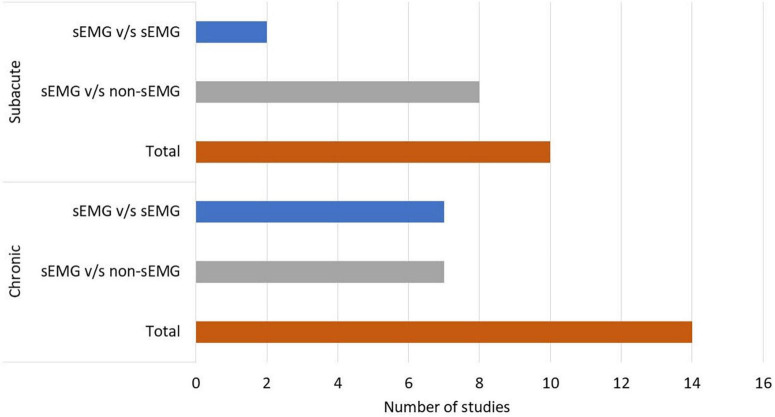
Number of studies performed in patients with chronic and subacute stroke. s-EMG v/s sEMG, studies comparing different surface electromyography driven interventions; s-EMG v/s non-sEMG, studies comparing surface electromyography driven interventions with non-surface electromyography driven groups.

**FIGURE 3:**
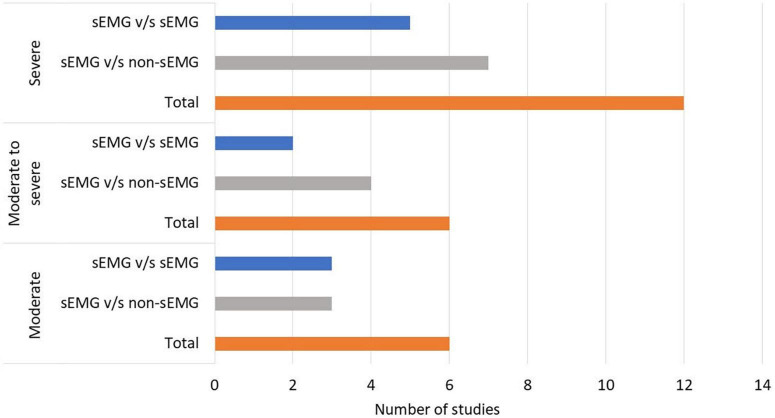
Number of studies performed in patients with moderate to severe upper limb impairment. s-EMG v/s sEMG, studies comparing different surface electromyography driven interventions; s-EMG v/s non-sEMG, studies comparing surface electromyography driven interventions with non-surface electromyography driven groups.

**FIGURE 4:**
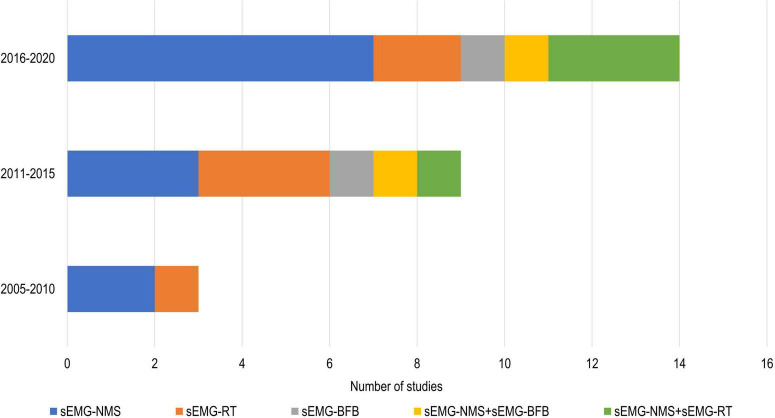
Number of studies using different sEMG interventions shown over time. s-EMG, surface electromyography; sEMG-NMS, sEMG triggered neuromuscular stimulation; sEMG-RT, s-EMG driven robot therapy; sEMG-BFB, sEMG providing visual and/or auditory biofeedback; +, combine with.

In total, 808 participants from 24 studies were included. The mean age of the included study groups, as reported by RCTs, varied between 42 and 72 years. The sample sizes varied from 6 to 51 participants for each intervention group. The participants were included in the chronic (*n* = 13), subacute (*n* = 8), mixed subacute/chronic (*n* = 2), or acute/subacute (*n* = 1) stage of stroke recovery (Figure 2). According to the total FMA-UE score, 12 studies included participants with severe, 6 with severe to moderate, and 6 with moderate upper extremity impairment (Figure 3). More than half of the studies (*n* = 14) were conducted in hospital settings, followed by research laboratory (*n* = 4), home (*n* = 4), and mixed home and hospital setting (n = 1). One study did not report their treatment location (Huang et al., 2020). Half of the studies (*n* = 12) used sEMG to trigger neuromuscular stimulation, 6 used sEMG to drive robotic devices, 2 to provide visual and/or auditory biofeedback, and 6 studies combined different types of sEMG interventions (Figure 4). As illustrated in Figure 4, the number of publications evaluating the single or combined effect of sEMG interventions on upper extremity function has increased over the past years.

The average dose of the interventions, as reported in the included studies, was 27 sessions provided 4 times per week for about 54 min per session (Tables 3, 4). However, one study provided a high-intensity training 7 times a week, 3 times a day for 30 min each time (de Kroon and IJzerman, 2008), and another study (Obayashi et al., 2020) had an individualized dose protocol varying from 3 to 35 sessions depending on the participant.

Half of the studies (*n* = 12) (Hemmen and Seelen, 2007; de Kroon and IJzerman, 2008; Hu et al., 2009, 2015; Singer et al., 2013; Amasyali and Yaliman, 2016; Kwakkel et al., 2016; Wilson et al., 2016; Qian et al., 2017, 2019; Mugler et al., 2019; Huang et al., 2020) reported follow-up data, and only two trials (Kwakkel et al., 2016; Wilson et al., 2016) reported more than one follow-up session. A follow-up at 3 months after the intervention was the most common. Only two studies (Amasyali and Yaliman, 2016; Kwakkel et al., 2016) reported a decline of the main outcome at follow-up. The sEMG signal source was commonly obtained from muscles at the wrist (*n* = 15), followed by both elbow and wrist (*n* = 5) (Hemmen and Seelen, 2007; de Kroon and IJzerman, 2008; Hu et al., 2009; De Araújo et al., 2011; Huang et al., 2020), elbow (*n* = 2) (Page et al., 2013, 2020), and all parts of the upper limb (*n* = 2) (Mugler et al., 2019; Obayashi et al., 2020). Half of the studies (*n* = 12) (De Araújo et al., 2011; Shindo et al., 2011; Cordo et al., 2013; Page et al., 2013, 2020; Singer et al., 2013; Kim et al., 2016; Kwakkel et al., 2016; Qian et al., 2017, 2019; Schick et al., 2017; Mugler et al., 2019) incorporated functional task to their sEMG training protocol, such as, grasp and release objects (Singer et al., 2013; Qian et al., 2017), pushing objects (Kim et al., 2016), virtual reality training (Kwakkel et al., 2016; Mugler et al., 2019), daily life activities (Shindo et al., 2011), and others (Page et al., 2013; Qian et al., 2019). The majority of the studies (*n* = 20) reported statistically significant improvements in FMA-UE scores compared to the baseline scores (Tables 3, 4).

### Meta-Analysis

#### Surface Electromyography Interventions in Comparison With Non-sEMG Interventions

**FIGURE 5:**
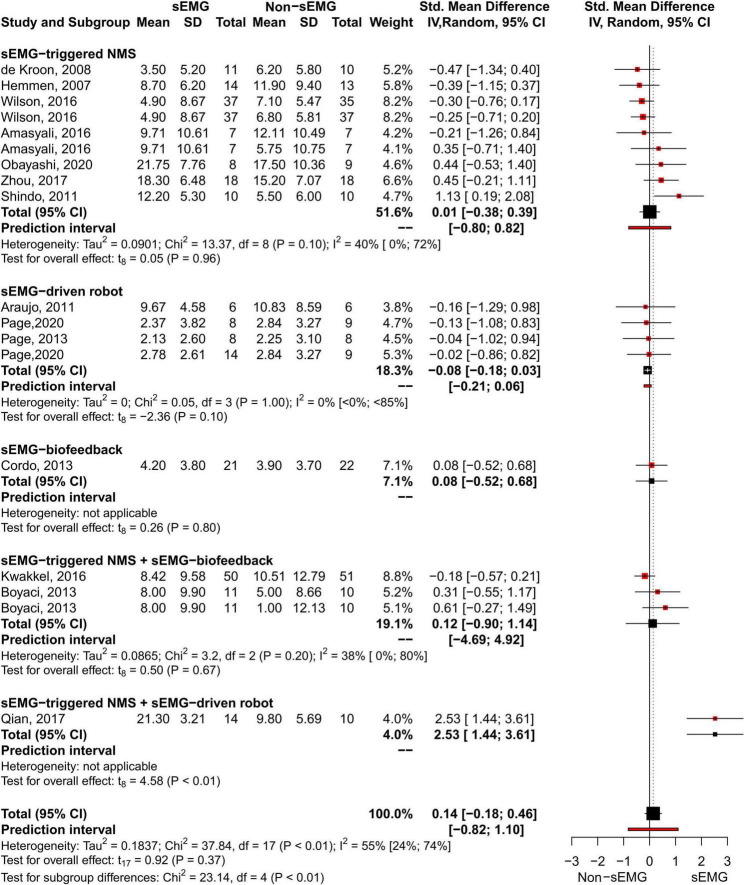
Meta-analyses of the effect of sEMG intervention versus non-sEMG intervention on total FMA-UE score. s-EMG, surface electromyography; NMS, neuromuscular stimulation; +, combine with.

The meta-analysis revealed no differences for the total effect of sEMG interventions compared to non-sEMG interventions on the total FMA-UE score (14 studies, 509 participants, SMD 0.14, P 0.37, 95% CI –0.18 to 0.46, I^2^ 55%, Figure 5). Moderate heterogeneity was present in the total pooled analysis (Higgins et al., 2019). No difference was found at the subgroup level when comparing the different types of sEMG interventions with non-sEMG interventions. Two studies, one that utilized sEMG-NMS (Shindo et al., 2011) and one that combined sEMG-NMS with sEMG-driven robotic device (Qian et al., 2017), showed a statistically significant effect on improving upper limb function compared to a non-sEMG intervention.

#### sEMG Intervention in Comparison With Another sEMG Interventions

**FIGURE 6:**
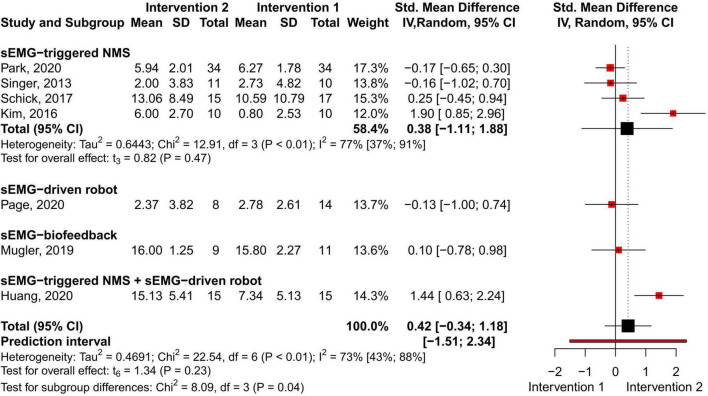
Meta-analyses of the effect of sEMG intervention versus another sEMG intervention on total FMA-UE score. s-EMG, surface electromyography; NMS, neuromuscular stimulation; +, combine with.

No difference in the overall and subgroup effect according to the total FMA-UE score was found in the meta-analysis comparing training protocols including sEMG interventions alone (Intervention 1) to protocols combining sEMG interventions with another training modality (Intervention 2) (7 studies, 213 participants, SMD 0.42, P 0.23, 95% CI –0.34 to 1.18, I^2^ 73%, Figure 6). Substantial heterogeneity was found for the overall result (Higgins et al., 2019). Two studies (Kim et al., 2016; Huang et al., 2020) showed an effect favoring Intervention 2. One of the studies (Huang et al., 2020) compared sEMG-driven robotic intervention alone to sEMG-driven robotic combined with sEMG-NMS, and the other study (Kim et al., 2016) compared sEMG-NMS to sEMG-NMS combined with task-oriented training. Moreover, the most common type of sEMG intervention in both meta-analyses was sEMG-NMS intervention.

## Discussion

The present systematic review and meta-analysis synthesizes the effects of different surface electromyography-driven interventions on upper limb function in people with stroke. A total of 24 studies (*n* = 808) were included in the systematic review, and 20 studies were included in the meta-analyses. The results of the meta-analyses revealed no differences for the total effect of sEMG interventions compared to non-sEMG interventions nor for studies comparing sEMG interventions with another sEMG intervention protocol when assessed by FMA-UE total score. Moreover, it is worth to notice that sEMG is not an intervention used in solitary but a tool to complement and enhance treatment effects of different neuromuscular rehabilitation interventions.

To the best of our knowledge, this is the first systematic review and meta-analysis summarizing the different sEMG-driven interventions utilized for upper limb rehabilitation in people with stroke. Earlier systematic reviews and meta-analyses have evaluated a single type of sEMG intervention, most commonly sEMG-NMS (Bolton et al., 2004; Meilink et al., 2008; Eraifej et al., 2017; Monte-Silva et al., 2019). Even though we included several types and protocols of sEMG interventions in the current review, the results could not verify which single or combined sEMG-intervention (combined with other sEMG modalities or with another training modality) was most beneficial for upper limb rehabilitation. In one of the meta-analyses, two studies (Shindo et al., 2011; Qian et al., 2017) showed an effect favoring the sEMG intervention over the non-sEMG intervention, and in the other meta-analysis, two studies (Kim et al., 2016; Huang et al., 2020) showed an effect favoring the sEMG intervention combined with another training modality compared to sEMG intervention alone. Furthermore, all included studies had a relatively small sample sizes (6 to 51 participants per group), the number of comparisons available for subgroup analysis was low (1 to 9 studies), and moderate to substantial heterogeneity was observed. All these factors need to be considered when interpreting the results.

A recent meta-analysis by Monte-Silva et al. (2019), evaluating the effect of sEMG-NMS interventions, showed an effect favoring sEMG-NMS compared to a control group (non-sEMG interventions) for improving upper limb function in people with stroke. In contrast to our results, showing no overall effect assessed by FMA-UE, their meta-analysis included studies using different outcome measures on upper limb function. Another significant difference from our analysis was that the mean difference for the overall effect was calculated between the post-intervention scores and not as a change from the baseline. This means that existing differences between groups at baseline [e.g., 40, 44, 53] were ignored in their analysis. Furthermore, Monte-Silva et al. (2019) used a fixed-effect model, which assumes that all included studies are similar enough and share a true effect size, which is highly unlikely taking the differences in the populations. In our analysis, the change from baseline and a random-effect model was used to estimate the overall effect, which might be the reason for contrasting results. A meta-analysis by Meilink et al. (2008) that included three studies, using FMA-UE as an outcome measure in their subgroup analysis, found no statistically significant differences in upper limb function between sEMG-NMS stimulation and conventional care. Moreover, an earlier meta-analysis by Bolton et al. from 2004 (Bolton et al., 2004), including three RCT and two non-RCT studies with small sample sizes, showed an overall beneficial effect of sEMG-NMS on arm and hand function measured with different clinical scales. Taken the varying results, it can be concluded that sEMG-NMS interventions might have a beneficial effect on improving arm and hand function as measured by FMA-UE, but this effect is not possible to verify when the sEMG interventions are compared to a non-sEMG interventions.

In the subgroup analysis of sEMG-driven robotic device interventions, no difference in the overall effect could be found. The sEMG-driven robotic device intervention has not previously been included in a meta-analysis, even though it has been considered as a potential rehabilitation option for stroke (Basteris et al., 2014). The number of studies using sEMG-driven robotic device has been increasing in the past years, which is promising, especially considering its potential to provide unsupervised intensive and repetitive training (Norouzi-Gheidari et al., 2012). However, the costs and complexity of robot devices might be a barrier for implementation in clinical and home settings.

Two studies that investigated sEMG-visual and/or auditory biofeedback were included in the meta-analysis (Cordo et al., 2013; Mugler et al., 2019). The results showed no difference in effect when compared to a non-sEMG intervention or to a different sEMG intervention protocol. This is in contrast to a small meta-analysis employing older methodology by Schleenbaker *et al.* from 1993 (Schleenbaker and Mainous, 1993), in which the authors concluded that the sEMG-visual and/or auditory biofeedback can be an effective tool for neuromuscular relearning in stroke (Schleenbaker and Mainous, 1993). Thus, more studies utilizing sEMG-visual and/or auditory biofeedback are needed to determine its effectiveness compared to other interventions.

Among the studies that combined different types of sEMG interventions, two studies (Qian et al., 2017; Huang et al., 2020) combining sEMG-NMS with sEMG-driven robotic devices, showed a favorable effect compared to non-sEMG intervention or sEMG-driven robotic device used alone, respectively. Even when these results are promising, more RCTs using sEMG interventions in combination with other interventions are needed to obtain its effect.

About half of the included studies in this systematic review incorporated functional tasks in their sEMG training protocol. This is in line with current literature which indicates that task-oriented training facilitates motor control, motor learning and brain activation patterns (Hubbard et al., 2009) and, therefore, might have positive impact on upper limb rehabilitation. The results also show that improvements in upper limb function can still be achieved in the chronic phase of stroke (De Araújo et al., 2011; Hu et al., 2015; Kim et al., 2016; Qian et al., 2019; Huang et al., 2020) and that sEMG-driven interventions might be feasible for people with severe upper limb impairment (Qian et al., 2017; Zhou et al., 2017; Huang et al., 2020). This is promising when considering the limited rehabilitation options available for people with chronic and severe upper limb impairments.

Most commonly, the training sessions lasted for 30 min and were applied 3 to 5 times a week, which could be considered as clinically feasible. Only a few studies included a follow-up assessment on their rehabilitation protocol, and therefore an evaluation of long-term effects are not feasible. Furthermore, most of the studies were conducted in the hospital or laboratory settings, and only a few were performed in a home environment. Since the home-based treatments can have several benefits, such as improved adherence, higher dose, and lower cost (Chaiyawat et al., 2009), future studies are needed to evaluate their effect and usefulness for stroke rehabilitation.

### Strengths and Limitations

The present systematic review and meta-analysis is, to our knowledge, the first to summarize and analyze the effects of different sEMG-driven interventions utilized for stroke upper limb rehabilitation. Even when the number of identified randomized clinical trials using sEMG-NMS can be considered sufficient in this meta-analysis, the number of studies included in the subgroup analysis was small. Another limitation is the relatively small sample sizes of the included studies. Only one study included 100 participants (Kwakkel et al., 2016), while the majority ranged between 12 to 40 participants. Moreover, substantial to moderate heterogeneity was found on the meta-analyses due to the high methodological variance occurring in the included studies. Also, it needs to be noticed that a variety of non-sEMG interventions were used as a comparison group in the included studies. Finally, it needs to be taken into account that six studies, with “fair” methodological quality according to the PEDro scale, were also included in the meta-analyses.

### Clinical Implications

This systematic review and meta-analysis found insufficient evidence to identify which types of sEMG-driven interventions have a beneficial effect on upper limb function compared to non-sEMG interventions. However, most of the sEMG interventions showed a positive effect on upper limb function post-intervention compared to baseline. From a clinical perspective, the positive effect observed in individuals with chronic and severe stroke impairment is promising and calls for further studies in these clinical subgroups.

## Conclusion

This review and meta-analysis synthesized the current evidence of different types of sEMG-driven interventions for upper limb rehabilitation in people with stroke. No differences in the total effect (measured by total FMA-UE score) of sEMG interventions compared to non-sEMG interventions, nor sEMG interventions compared to sEMG interventions combined with other therapies, were found in our meta-analyses. Although we included several types of sEMG-driven interventions, the results could not verify which single or combined sEMG-intervention was most beneficial for upper limb function. Notably, most of the included studies reported statistically significant improvements on upper limb function compared to baseline FMA-UE scores, and these effects were shown even on participants with severe stroke in the chronic stage of stroke recovery.

Even though, surface EMG-driven interventions are promising for promoting upper limb function in people with stroke, more randomized controlled trials with larger sample sizes, unified methodology and outcome measures are required to establish the effectiveness of the different types of sEMG interventions in combination with other therapies. Considering the high-intensity training that sEMG interventions can offer, future studies should further explore its effectiveness in home settings, especially in chronic stroke cases.

## Data Availability Statement

The original contributions presented in the study are included in the article/supplementary material, further inquiries can be directed to the corresponding author/s.

## Author Contributions

MAM, MM-N, MBK, and AN organized the database. MAM, MM-N, and MBK screened the included studies. MM-N performed the meta-analysis and wrote the first draft of the manuscript. All authors contributed to conception and design of the study, manuscript revision, read, and approved the submitted version.

## Conflict of Interest

The authors declare that the research was conducted in the absence of any commercial or financial relationships that could be construed as a potential conflict of interest.

## Publisher’s Note

All claims expressed in this article are solely those of the authors and do not necessarily represent those of their affiliated organizations, or those of the publisher, the editors and the reviewers. Any product that may be evaluated in this article, or claim that may be made by its manufacturer, is not guaranteed or endorsed by the publisher.
